# GABA in Paraventricular Nucleus Regulates Adipose Afferent Reflex in Rats

**DOI:** 10.1371/journal.pone.0136983

**Published:** 2015-08-28

**Authors:** Lei Ding, Run Gao, Xiao-Qing Xiong, Xing-Ya Gao, Qi Chen, Yue-Hua Li, Yu-Ming Kang, Guo-Qing Zhu

**Affiliations:** 1 Key Laboratory of Cardiovascular Disease and Molecular Intervention, Department of Physiology, Nanjing Medical University, Nanjing, Jiangsu 210029, China; 2 Department of Pathophysiology, Nanjing Medical University, Nanjing, Jiangsu 210029, China; 3 Department of Physiology and Pathophysiology, Cardiovascular Research Center, Xi'an Jiaotong University School of Medicine, Xi'an 710061, China; University of Sydney, AUSTRALIA

## Abstract

**Background:**

Chemical stimulation of white adipose tissue (WAT) induces adipose afferent reflex (AAR), and thereby causes a general sympathetic activation. Paraventricular nucleus (PVN) is important in control of sympathetic outflow. This study was designed to investigate the role of γ-aminobutyric acid (GABA) in PVN in regulating the AAR.

**Methodology/Principal Findings:**

Experiments were carried out in anesthetized rats. Renal sympathetic nerve activity (RSNA) and mean arterial pressure (MAP) were continuously recorded. AAR was evaluated by the RSNA and MAP responses to electrical stimulation of the right epididymal WAT (eWAT) afferent nerve. Electrical stimulation of eWAT afferent nerve increase RSNA. Bilateral microinjection of the GABA_A_ receptor agonist isoguvacine or the GABA_B_ receptor agonist baclofen attenuated the AAR. The effect of isoguvacine on the AAR was greater than that of baclofen. The GABA_A_ receptor antagonist gabazine enhanced the AAR, while the GABA_B_ receptor antagonist CGP-35348 had no significant effect on the AAR. Bilateral PVN microinjection of vigabatrin, a selective GABA-transaminase inhibitor, to increase endogenous GABA levels in the PVN abolished the AAR. The inhibitory effect of vigabatrin on the AAR was attenuated by the pretreatment with gabazine or CGP-35348. Pretreatment with combined gabazine and CGP-35348 abolished the effects of vigabatrin.

**Conclusions:**

Activation of GABA_A_ or GABA_B_ receptors in the PVN inhibits the AAR. Blockade of GABA_A_ receptors in the PVN enhances the AAR. Endogenous GABA in the PVN plays an important role in regulating the AAR.

## Introduction

White adipose tissues (WAT) are innervated by both sensory fibers and efferent sympathetic fibers [1,2]. The efferent and afferent neural innervation of WAT are involved in body fat regulation [3,4]. Chemical stimulation of white adipose tissues (WAT) with capsaicin, bradykinin, adenosine or leptin induces sympathetic activation [5,6]. The sympatho-excitatory reflex, adipose afferent reflex (AAR) may be important in promoting lipolysis and energy expenditure [2,6,7]. We have found that the AAR is enhanced in obesity and the enhanced AAR partially contributes to the excessive sympathetic activation and hypertension in obesity [8]. Hypothalamic paraventricular nucleus (PVN) is an important integrative site within the brain in control of sympathetic outflow and cardiovascular activity [9]. The AAR is abolished by the inhibition of the PVN with lidocaine or lesion of PVN neurons with kainic acid, indicating the importance of the PVN in the control of AAR [5,8].

Ionotropic glutamate receptors in the PVN mediate the AAR and regulate sympathetic outflow in rats [10]. The activation of ionotropic glutamate receptors in the PVN is involved in the AAR-induced production of superoxide anions, and NAD(P)H oxidase-derived superoxide anions in the PVN contributes to the tonic modulation of AAR [11]. Furthermore, activation of melanocortin-4 receptors in the PVN enhances the AAR via a cAMP-PKA pathway [12]. It is known that γ-aminobutyric acid (GABA) in the PVN reduces blood pressure and sympathetic outflow [13,14]. Ionotropic GABA_A_ receptors and metabotropic GABA_B_ receptors are involved in regulating blood pressure and sympathetic outflow in normal and hypertensive rats [15]. The present study is designed to determine whether GABA in the PVN plays an inhibitory role in modulating AAR.

## Materials and Methods

Experiments were performed in 125 male Sprague–Dawley rats weighing between 350 and 400 g. The rats were caged in a controlled temperature and humidity with a 12-hour light/dark cycle with free access to standard laboratory chow and drinking water. The experiments were approved by the Experimental Animal Care and Use Committee of Nanjing Medical University and complied with the Guide for the Care and Use of Laboratory Animals (NIH publication, 8th edition, 2011).

### General procedures

The rat was anesthetized with intraperitoneal injection of urethane (800 mg/kg) and α-chloralose (40 mg/kg). The depth of anesthesia was determined by the absence of corneal reflexes and paw withdrawal response to a noxious pinch. The rat was ventilated with room air using a rodent ventilator (683, Harvard Apparatus Inc, USA). The right carotid artery was cannulated for continuously recording of blood pressure. The rats were allowed to stabilize for 30–60 min after surgery. At the end of the experiment, the rats were euthanized with an overdose of pentobarbital sodium (150 mg/kg, iv).

### PVN microinjection

PVN microinjections were carried out as we previously reported [16,17]. Stereotaxic coordinates for PVN were 1.8 mm caudal from bregma, 0.4 mm lateral to the midline and 7.9 mm ventral to the dorsal surface. Bilateral PVN microinjections were carried out with two glass micropipettes (about 50 μm tip diameter) and completed within 1 min. The microinjection volume was 50 nl for each side of the PVN [11]. The effective doses of chemicals used for the PVN microinjection were based upon previous studies [14,18–21]. At the end of the experiment, 50 nl of Evans blue was injected into each microinjection site for histological identification of the microinjection sites. Total 5 rats scattered in different groups were excluded from data analysis because the microinjection sites were outside one side of the PVN.

### Recording of renal sympathetic nerve activity

Renal sympathetic nerve activity (RSNA) were recorded as we previously reported [10,22]. In brief, a left retroperitoneal incision was made, and the left renal nerve was isolated, and cut distally to eliminate its afferent activity. A pair of silver electrodes was placed on the central end of the nerve and immersed in warm mineral oil. The signals were amplified with a four-channel AC/DC differential amplifier (DP-304, Warner Instruments, Hamden, CT, USA) with a high pass filter at 10 Hz and a low pass filter at 3,000 Hz, and was integrated at a time constant of 100 ms. The RSNA and mean arterial pressure (MAP) were simultaneously recorded with a PowerLab data acquisition system (8/35, ADInstruments, Castle Hill, Australia). Background noise was determined after section of the central end of the renal sympathetic nerve at the end of the experiment, and was subtracted from the integrated values of the RSNA.

### Evaluation of AAR

An incision was made on the skin of right scrotum to expose the epididymal WAT (eWAT). A drop of 1% toluidine blue was applied to the fat pad to facilitate visualization of the nerves. One of the right eWAT afferent nerves was isolated, and cut distally. A pair of silver electrodes was placed on the central end of the nerve and immersed in warm mineral oil. The stimulation electrodes were connected to the stimulator via an isolator. The pulse width of stimulation was kept at 1 ms and each stimulus lasted 1 min. The frequency and voltage of stimulus were set as 30 Hz and 5V in the present study except the experiment for determining the effects of different frequency and voltage stimulation on the AAR. The AAR was evaluated by the RSNA and MAP responses to electrical stimulation. At the end of the experiment, the reliability of the stimulation of eWAT afferent nerve was confirmed by no RSNA and MAP response to the stimulation after section the central end of the nerve.

Capsaicin-induced AAR was conducted as we previously reported [8,10,23]. Simply, right inguinal WAT (iWAT) was exposed, and four stainless steel tubes (0.31 mm outer diameter) were inserted into the fat pad 3 mm below the surface of the fat pads. The tubes were 4 mm apart from each other. The AAR was induced by infusion of capsaicin (1.0 nmol/μL) into four sites of the right iWAT at a rate of 4.0 μL/min for 2 min for each site. The AAR was evaluated by the RSNA and MAP responses to capsaicin.

### Experimental protocols

Firstly, the electrical stimulation-induced AAR was identified in four groups of rats (n = 6 for each). (1) Six rats randomly received different frequency of stimulation (sham stimulation, 3, 10, 30 or 60 Hz) with a voltage of 5 V to determine the effects of different stimulation frequency on RSNA and MAP; (2) Six rats randomly received different voltage of stimulation (sham stimulation, 1, 5, or 10 V) with a frequency of 30 Hz to determine the effects of different stimulation intensity on RSNA and MAP. We found that the stimulus at 30 Hz and 5 V almost caused a maximal RSNA and MAP responses, and thus was selected for the present study. (3) Six rats randomly received sham stimulation, stimulation of eWAT artery, stimulation of peripheral end of eWAT nerve, or stimulation of central end of eWAT nerve to exclude the possibility that the stimulation may spread to other tissues; (4) Six rats randomly received sham stimulation, stimulation of central end of eWAT nerve, infusion of saline or capsaicin into iWAT to compare the electrical stimulation-induced AAR with capsaicin-induced AAR. The interval between each intervention for complete recovery was at least 20 min for electrical stimulation-induced AAR, and at least 60 min for capsaicin-induced AAR. (5) Six rats received sham stimulation and electrical stimulation (5 V, 30 Hz) of eWAT afferent nerves 4 times at interval of 20 min to determine the efficiency of repetition stimulation-evoked AAR in the same rat.

Secondly, effects of PVN microinjection of different doses of a GABA_A_ receptor agonist isoguvacine and a GABA_B_ receptor agonist baclofen on the AAR were determined in two groups of rats (n = 6 for each). Six rats were randomly subjected to the PVN microinjection of saline or isoguvacine (0.1, 1.0 or 10.0 nmol), and another six rats were subjected to the PVN microinjection of saline or baclofen (0.1, 1.0 or 10.0 nmol). The interval between each intervention was at least 60 min for complete recovery. The AAR induced by electrical stimulation was evaluated 20 min after each microinjection.

Thirdly, effects of PVN microinjection of a GABA_A_ receptor antagonist gabazine and a GABA_B_ receptor antagonist CGP35348 on the AAR were determined in eight groups of rats (n = 6 for each). One group of rats (control) received the PVN microinjection of saline followed by sham electrical stimulation. Other seven groups of rats received the PVN microinjection of saline, isoguvacine (10 nmol), gabazine (0.1 nmol), gabazine+isoguvacine, baclofen (10 nmol), CGP35348 (10 nmol) or CGP35348+baclofen followed by electrical stimulation. Gabazine or CGP35348 was administered 10 min before isoguvacine or baclofen, respectively. The AAR induced by electrical stimulation was evaluated 20 min after the completion of the PVN microinjection.

Lastly, the effects of PVN microinjection of a GABA-transminase inhibitor vigabatrin on the AAR were determined in six groups of rats (n = 6 for each). One group of rats (control) received the PVN microinjection of saline followed by sham electrical stimulation. Other five groups of rats received the PVN microinjection of saline, vigabatrin (10 nmol), gabazine (0.1 nmol)+vigabatrin, CGP35348 (10 nmol)+vigabatrin or (gabazine+CGP35348)+vigabatrin followed by electrical stimulation. Gabazine, CGP35348 or gabazine+CGP35348 was administered 10 min before vigabatrin. The AAR induced by electrical stimulation was evaluated 20 min after the completion of the PVN microinjection.

### Chemicals

Isoguvacine, baclofen, gabazine, CGP-35348 and vigabatrin were obtained from Sigma Chemical Co. All these chemicals were dissolved in normal saline.

### Statistics

Baseline RSNA or MAP were determined by averaging 2 min of its maximal responses within the time range from 5 min to 10 min after the PVN microinjection. AAR was evaluated by averaging 30 s of the maximal RSNA and MAP responses to electrical stimulation within the time range from 10 s to 50 s after the beginning of the electrical stimulation. Comparisons between two observations in the same animal were assessed by Student’s paired t test. The difference between groups were determined with a one-way or two-way ANOVA followed by the Newman-Keuls test for post hoc analysis of significance. All data were expressed as mean ± SE. A value of P<0.05 was considered statistically significant.

## Results

### AAR induced by different frequency and intensity of electrical stimulation

**Fig 1:**
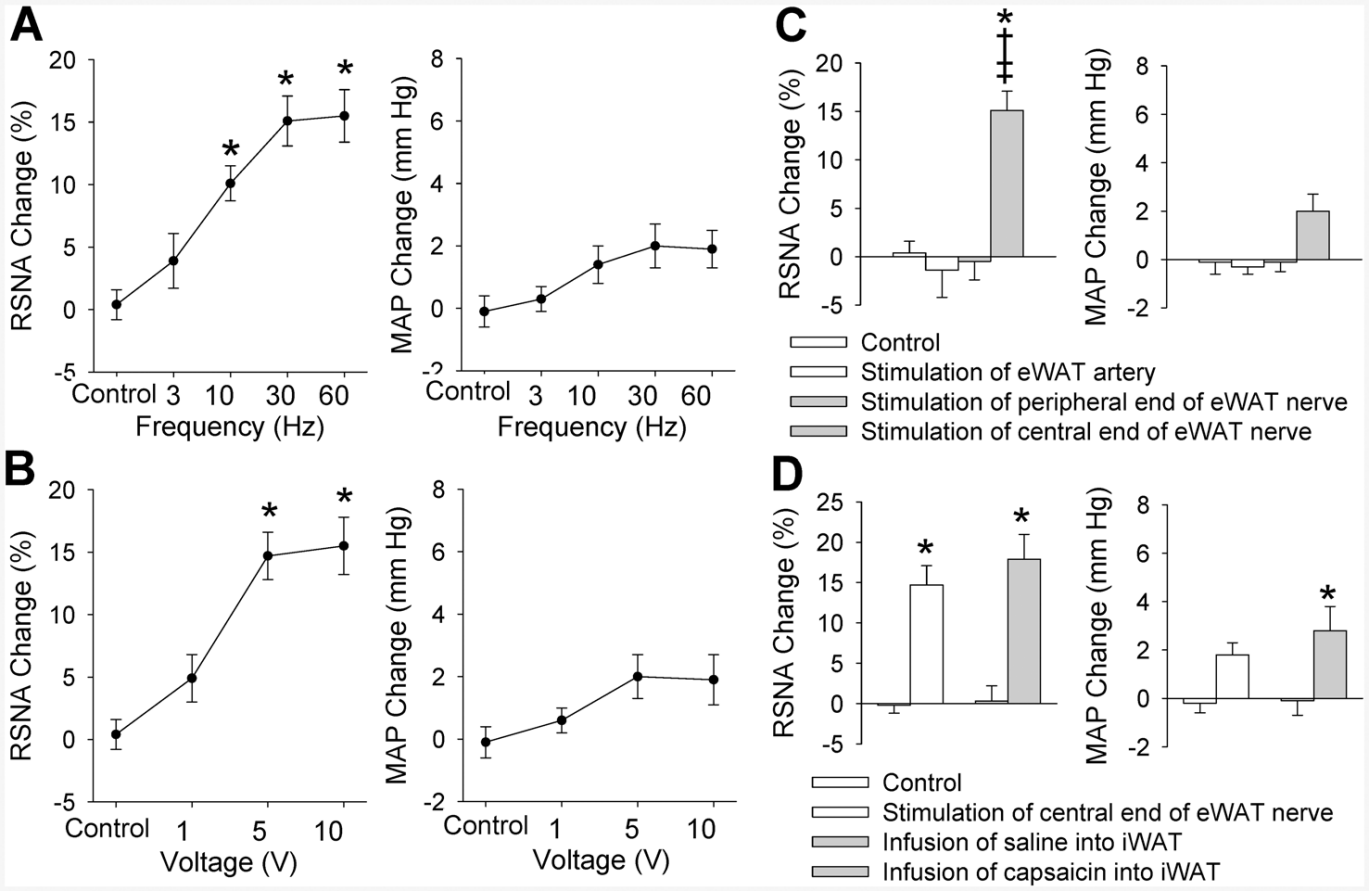
AAR induced by electrical stimulation of right eWAT. A, effects of different frequency of stimulation (0, 3, 10, 30 and 60 Hz, 5V) on the RSNA and MAP; B, effects of different voltage of stimulation (0, 1, 5 and 10 V, 30 Hz) on the RSNA and MAP; C, stimulation of the peripheral end of the right eWAT or the WAT artery had no significant effect on the RSNA and MAP. D, difference between the AAR induced by stimulation of central end of eWAT nerve and the AAR induced by infusion of capsaicin into the iWAT. Control means sham electrical stimulation. Values are mean±SE. * P<0.05 vs. Control; † P<0.05 vs. Stimulation of eWAT artery; ‡ P<0.05 vs. Stimulation of peripheral end of eWAT nerve. n = 6 for each group.

**Fig 2:**
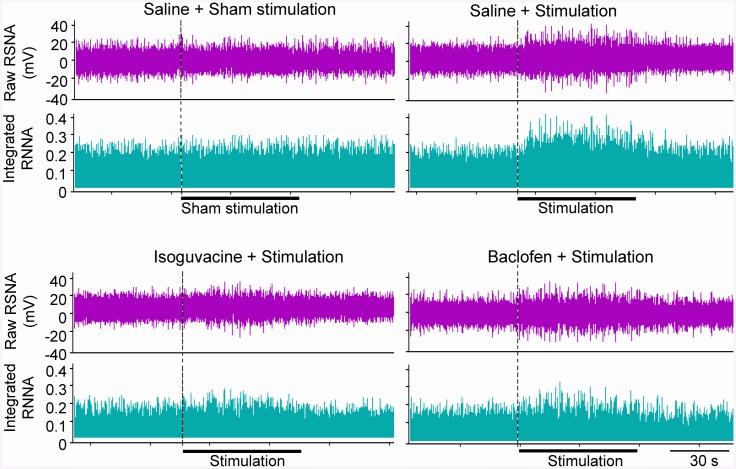
Representative traces showing the effects of pretreatment with the PVN microinjection of saline, isoguvacine (a GABA_A_ receptor agonist, 10 nmol) or baclofen (a GABA_B_ receptor agonist, 10 nmol) on the RSNA induced by electrical stimulation of right eWAT (30 Hz, 5 V, 1 min).

Electrical stimulation of the central end of the eWAT nerve at 10, 30 or 60 Hz with a constant voltage of 5 V caused a significant increase in RSNA (Fig 1A). Similarly, the stimulation at 5 or 10 V with a constant frequency of 30 Hz caused a significant increase in RSNA (Fig 1B). However, these stimulus only caused a tendency in increasing MAP, but the difference did not reach the significant level. The stimulus at 30 Hz and 5 V almost caused a maximal RSNA and MAP responses, and was selected for the present study. The RSNA response to electrical stimulation occurred in 1–3 s after the start of the stimulation, and disappeared in 5–10 s after the stimulation withdrawal (Fig 2). To exclude the possibility that the RSNA and MAP responses were caused by the stimulation diffusion to other tissues, we examined the effects of electrical stimulation of the peripheral end of the eWAT nerve or the eWAT artery on the RSNA and MAP. It was found that electrical stimulation of the peripheral end of the eWAT nerve or stimulation of the eWAT artery failed to induce any RSNA and MAP responses (Fig 1C). The AAR-induced by electrical stimulation of eWAT nerve was similar to the AAR-induced by iWAT infusion of capsaicin (Fig 1D). On the other hand, electrical stimulation (5 V, 30 Hz) of eWAT afferent nerves 4 times at interval of 20 min in the same rat induced similar degree of AAR, indicating the efficiency of repetition stimulation-evoked AAR (S1 Fig).

### Effects of GABA receptor agonist on AAR

**Fig 3:**
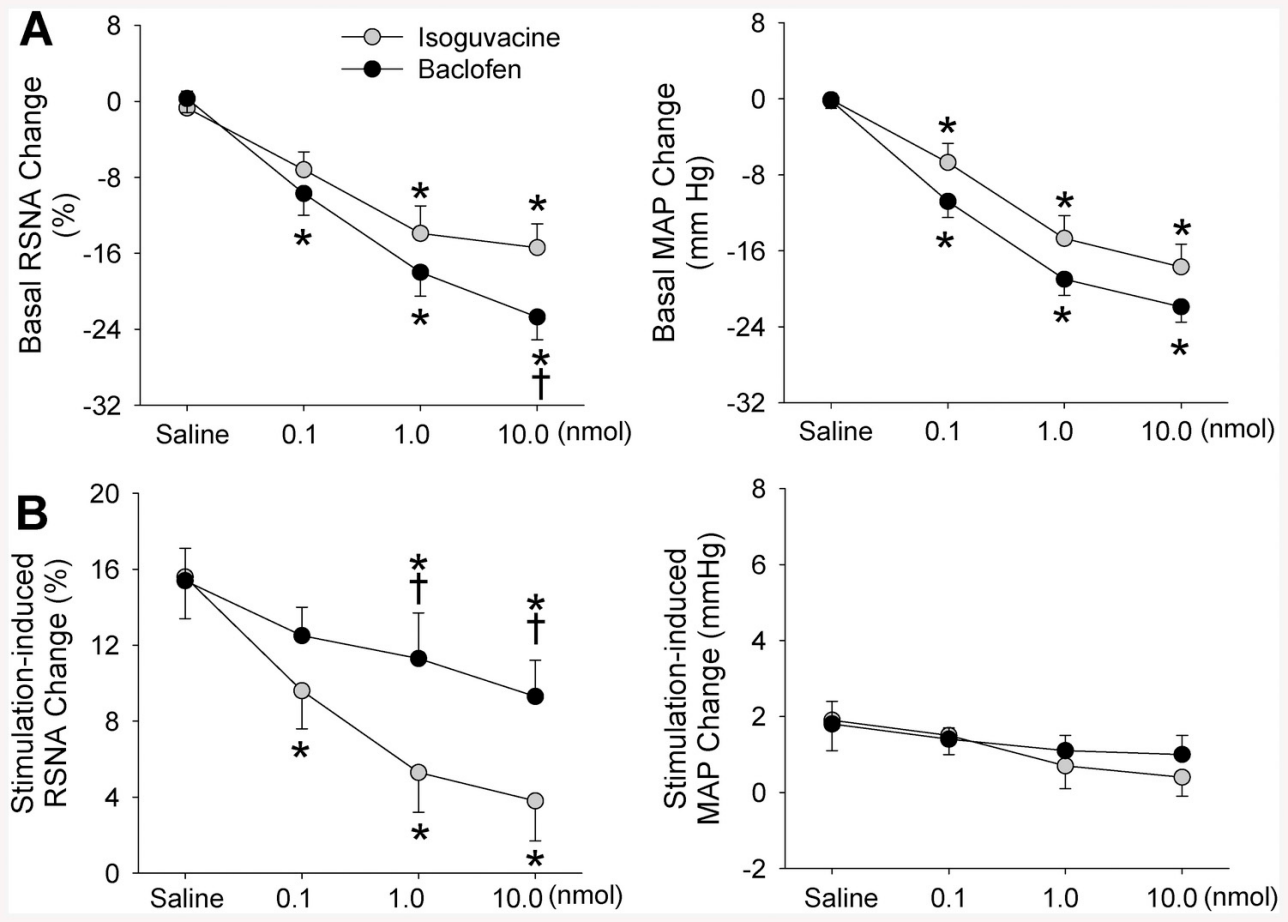
Effects of PVN microinjection of different doses of a GABA_A_ receptor agonist isoguvacine or a GABA_B_ receptor agonist baclofen (0.1, 1.0 or 10.0 nmol) on the basal RSNA and MAP, and the AAR induced by electrical stimulation of right eWAT. A, baseline RSNA and MAP changes; B, changes in electrical stimulation-induced AAR. Values are mean±SE. *P<0.05 vs. Saline; †P<0.05 vs. isoguvacine. n = 6 for each group.

PVN microinjection of a GABA_A_ receptor agonist isoguvacine or a GABA_B_ receptor agonist baclofen dose-dependently decreased baseline RSNA and MAP (Table 1). The role of high dose of isoguvacine in reducing baseline RSNA was smaller than that of baclofen (Fig 3A). Either isoguvacine or baclofen dose-dependently inhibited the AAR. However, the role of high doses of isoguvacine in inhibiting the RSNA response to electrical stimulation of eWAT afferent nerve was greater than that of baclofen (Fig 3B). Effects of microinjection of same dose of isoguvacine or baclofen into the anterior hypothalamic area, which is adjacent to the PVN, were determined to exclude the possibility that the effects of isoguvacine and baclofen were caused by diffusion to other brain area in rats (n = 3 for each group). It was found that isoguvacine or baclofen in the anterior hypothalamic area had no significant effects on the AAR (S2 Fig).

**Table 1:** Baseline RSNA (% of control) and MAP (mm Hg).

Groups	RSNA (% of control)		MAP (mm Hg)	
	Before	After	Before	After
Saline	100	100.2±1.1	90.0±3.9	90.2±4.0
Isoguvacine, 0.1 nmol	100	92.8±1.9* †	93.5±4.5	86.8±4.7
Isoguvacine, 1 nmol	100	86.1±2.9* †	91.6±3.5	76.9±2.6* †
Isoguvacine, 10 nmol	100	84.6±2.5* †	93.6±3.5	75.9±3.4* †
Baclofen, 0.1 nmol	100	90.3±2.3* †	89.1±3.9	78.3±3.1* †
Baclofen, 1 nmol	100	82.0±2.5* †	92.8±4.9	73.8±3.6* †
Baclofen, 10 nmol	100	77.3±2.4* †	92.0±3.6	70.2±4.2* †
Gabazine, 0.1 nmol	100	117.8±2.7* †	90.9±5.1	113.7±6.7* †
CGP35348, 10 nmol	100	111.9±2.1* †	90.2±4.7	95.6±4.5
Vigabatrin, 10 nmol	100	68.6±3.8* †	93.1±4.3	71.5±2.8* †

The RSNA data before intervention were taken as 100%. Values are mean±SE.

*P<0.05 compared with the value before intervention.

^†^P<0.05 compared with Saline. n = 6 for each group.

### Effects of GABA receptor antagonist on AAR

**Fig 4:**
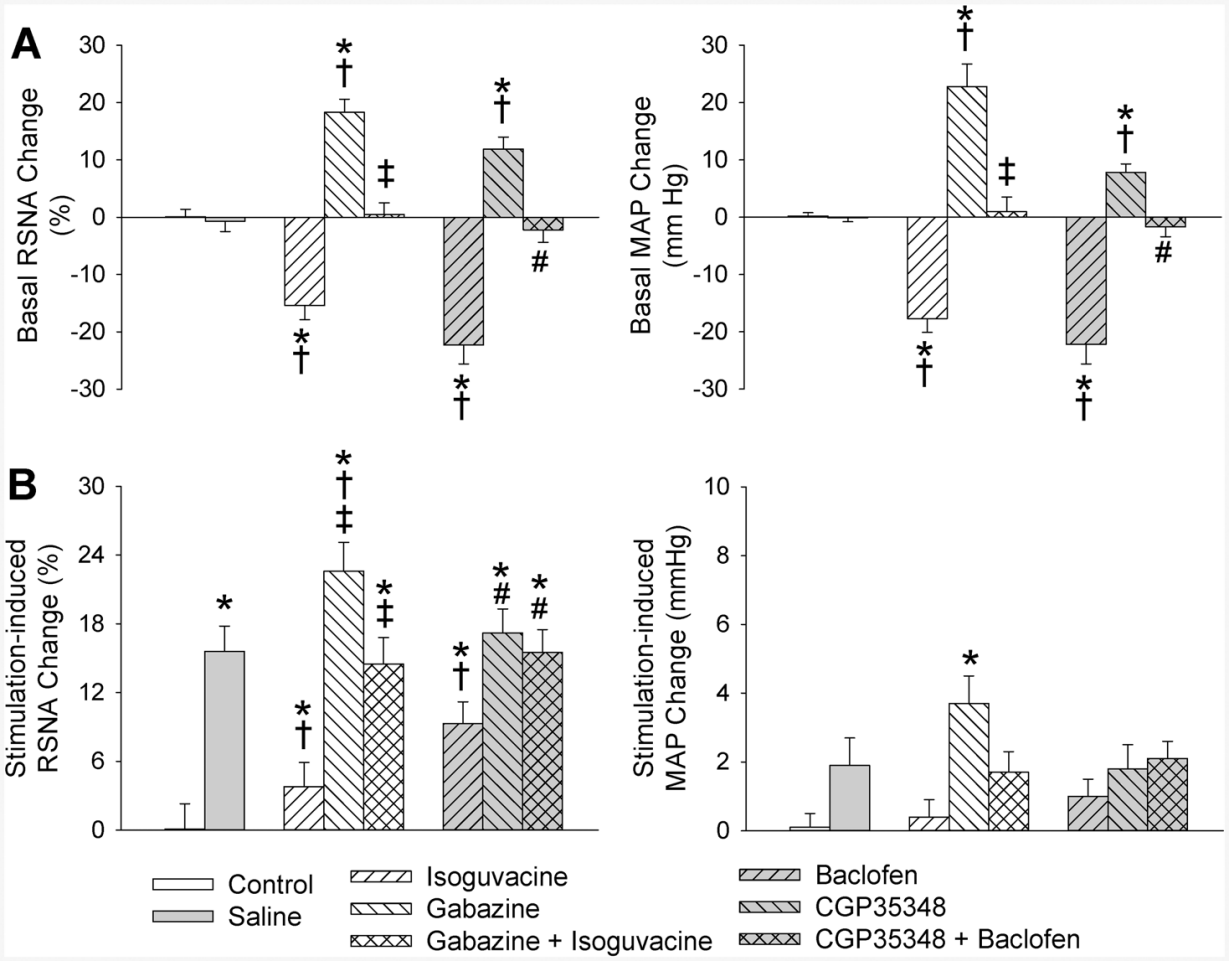
Effects of the PVN microinjection of gabazine (a GABA_A_ receptor antagonist, 0.1 nmol) or CGP35348 (a GABA_B_ receptor antagonist, 10 nmol) on the basal RSNA and MAP, and the AAR induced by electrical stimulation of right eWAT. A, baseline RSNA and MAP changes; B, changes in electrical stimulation-induced AAR. Control rats received the PVN microinjection of saline and sham electrical stimulation. Gabazine or CGP35348 was administered 10 min before isoguvacine (a GABA_A_ receptor agonist, 10 nmol) or baclofen (a GABA_B_ receptor agonist, 10 nmol). Electrical stimulation of right eWAT was carried out 20 min after the PVN microinjection. Values are mean±SE. *P<0.05 vs. Control; †P<0.05 vs. Saline; ‡P<0.05 vs. isoguvacine; #P<0.05 vs. baclofen. n = 6 for each group.

PVN microinjection of a GABA_A_ receptor antagonist gabazine or a GABA_B_ receptor antagonist CGP35348 increased baseline RSNA and MAP, and the effects of gabazine were greater than that of CGP35348 (Fig 4A). Gabazine enhanced the AAR, while CGP35348 had no significant effect on the AAR. Isoguvacine inhibited the AAR, which were abolished by the pretreatment with gabazine. Similarly, baclofen inhibited the AAR, which were abolished by the pretreatment with CGP35348 (Fig 4B).

### Effects of GABA-transaminase inhibitor on AAR

**Fig 5:**
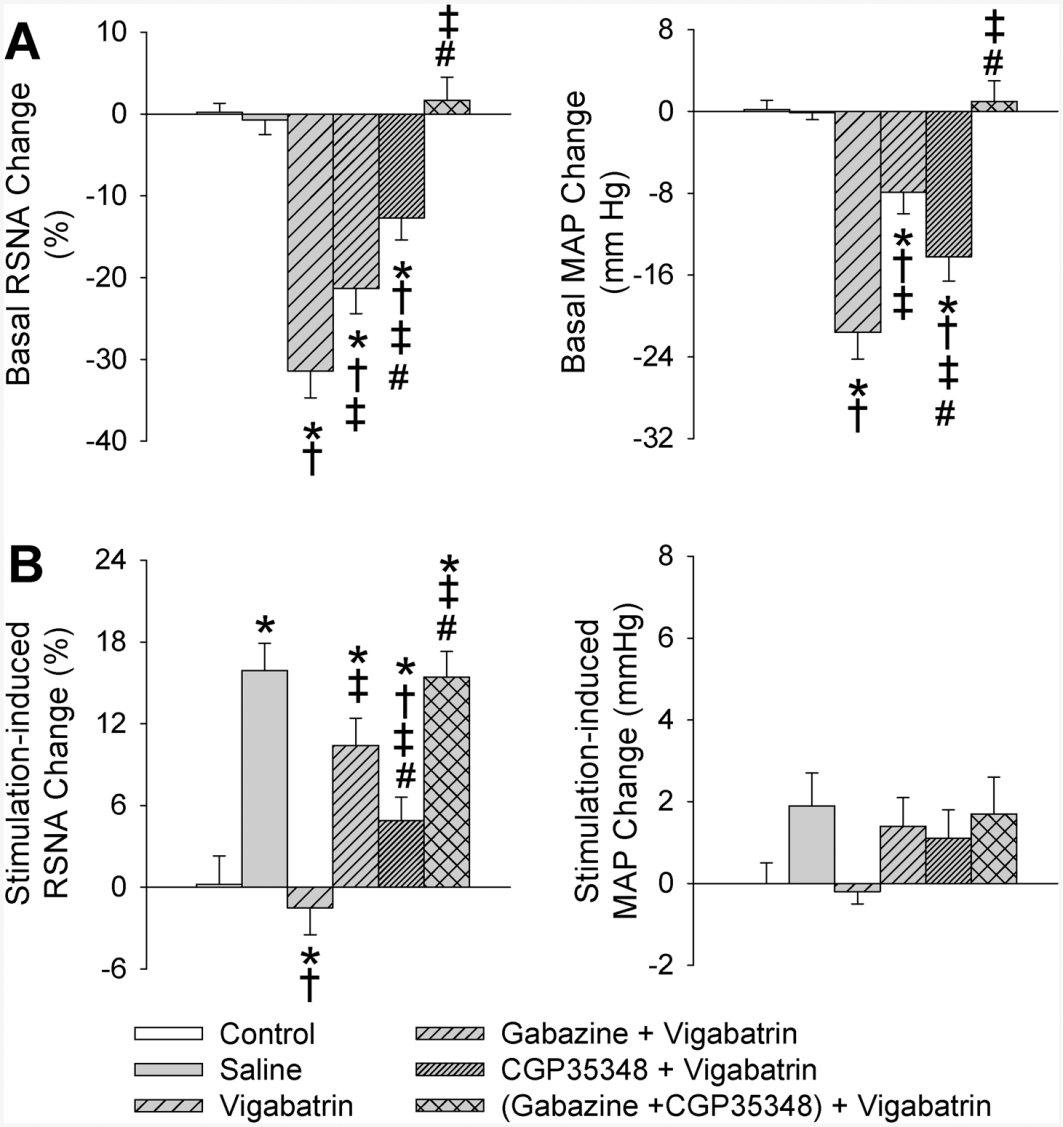
Effects of the PVN microinjection of saline, vigabatrin (a GABA-transminase inhibitor, 10 nmol) on the basal RSNA and MAP, and the AAR induced by electrical stimulation of right eWAT. A, baseline RSNA and MAP changes; B, changes in electrical stimulation-induced AAR. Control rats received the PVN microinjection of saline and sham electrical stimulation. Gabazine (a GABA_A_ receptor antagonist, 0.1 nmol), CGP35348 (a GABA_B_ receptor antagonist, 10 nmol) or combined gabazine and CGP35348 was administered 10 min before vigabatrin administration. Electrical stimulation of right eWAT was carried out 20 min after the PVN microinjection. Values are mean±SE. *P<0.05 vs. Control; †P<0.05 vs. Saline; ‡P<0.05 vs. Vigabatrin; #P<0.05 vs. Gabazine+Vigabatrin. n = 6 for each group.

Vigabatrin is a selective GABA-transaminase inhibitor and is used to increase endogenous GABA, which activates both GABA_A_ and GABA_B_ receptors [21,24]. PVN microinjection of vigabatrin reduced baseline RSNA and MAP, which were attenuated by GABA_A_ receptor antagonist gabazine or a GABA_B_ receptor antagonist CGP35348, and abolished by combined gabazine and CGP35348 (Fig 5A). More importantly, vigabatrin completely inhibited the electrical stimulation-induced AAR, and the effect of vigabatrin was attenuated by gabazine or CGP35348, and abolished by combined gabazine and CGP35348 (Fig 5B).

## Discussion

Previous studies in our lab have shown that ionotropic glutamate receptors in the PVN mediate the AAR [10], and activation of melanocortin-4 receptors in the PVN enhances the AAR [12]. The primary new findings in the present study are that activation of GABA_A_ or GABA_B_ receptors in the PVN inhibits the AAR, and blockade of GABA_A_ receptors in the PVN enhances the AAR. Increased endogenous GABA in the PVN completely inhibits the AAR via both GABA_A_ and GABA_B_ receptors. These results indicate the importance of GABA in the PVN in inhibiting the AAR.

PVN is an important brain region in regulating blood pressure and sympathetic outflow [9,23,25,26]. GABA in the PVN reduced blood pressure and sympathetic outflow [13,14] via both ionotropic GABA_A_ receptors and metabotropic GABA_B_ receptors [15]. It is known that isoguvacine, a selective GABA_A_ receptor agonist, induces an increase in membrane Cl^-^ conductance, causing neuronal hyperpolarization and inhibition via GABA_A_ receptors on postsynaptic neurons [27]. Baclofen, a selective agonist for GABA_B_ receptors, can modulate transmitter release by suppressing presynaptic Ca^2+^ channel, or cause a postsynaptic hyperpolarization by opening K^+^ channel via GABA_B_ receptors on the presynaptic and postsynaptic sites [28]. Vigabatrin is known to be a selective GABA-transaminase inhibitor which increases endogenous GABA [21,24]. We found that activation of GABA_A_ receptors in the PVN caused a more potent inhibitory effect on the AAR than the activation of GABA_B_ receptors. The endogenous GABA increase due to the inhibition of GABA-transminase in the PVN completely abolished the AAR, which were primarily mediated by GABA_A_ receptors and secondarily mediated by GABA_B_ receptors. On the other hand, the AAR was enhanced by the blockade of GABA_A_ receptors, but not by the blockade of GABA_B_ receptors. These results indicate that GABA_A_ receptors in the PVN are more important in regulating AAR than GABA_B_ receptors, and GABA_A_ receptors rather than GABA_B_ receptors play a tonic role in inhibiting the AAR.

It is known that baseline RSNA and MAP depends on complex central integration. Adipose afferent activity reflexly increases sympathetic outflow and blood pressure and will inevitably have an impact on the baseline RSNA and MAP. We noted that the effects of GABA_B_ receptor agonist were greater on baseline RSNA and MAP but smaller on the AAR than those of GABA_A_ receptor agonist, while where those of the antagonists are the opposite on the baseline and AAR. It is possible that the baseline RSNA and MAP are controlled by many factors although the AAR partially contributes to the baseline RSNA change. Activation of GABA_B_ receptors may not only inhibit the AAR, but cause more important inhibitory effects on other sympatho-excitatory mechanisms which are crucial for the baseline. Increased GABA release in the PVN will inhibit AAR and thus play a role in reducing baseline RSNA and MAP primarily via GABA_A_ receptors. Blockade of GABA_A_ receptors enhanced the AAR, suggesting that GABA_A_ receptors in the PVN are involved in the tonic regulation of baseline RSNA and MAP in physiological state. GABA_B_ receptor antagonist increase baseline but failed to enhance the AAR, suggesting that GABA_B_ receptors in the PVN is involved in the tonic inhibition of other sympatho-excitatory mechanisms rather than the AAR in physiological state.

Chemical stimulation of WAT with capsaicin, leptin, bradykinin or adenosine was used to induce AAR in our previous studies [5,8,11]. In this study, we introduced a new method to induce AAR with electrical stimulation of the WAT afferent nerve. The method was considered to be better in determining the central regulation of the AAR because the electrical stimulation-induced RSNA response was rapid and can be completely recovered in a short period. Furthermore, the AAR can be tested several times in each animal. This approach is in line with the guidelines of the National Centre for the Replacement, Refinement and Reduction of Animals in Research (London, UK). The eWAT afferent nerve was selected for electrical stimulation in the present study as the eWAT nerves are longer than the iWAT nerves, and easy to separate out enough length for the stimulation. However, the electrical stimulation of eWAT afferent nerve induced a significant increase in RSNA, but only a tendency in increasing the MAP and failed to reach statistical significance. Previous studies showed that chemical stimulation of WAT with capsaicin significantly increased both RSNA and MAP. It seemed that the MAP response to electrical stimulation of eWAT afferent nerve was smaller than that to chemical stimulation of iWAT with capsaicin reported in previous studies [5,6]. A possible reason may be that only one of eWAT afferent nerves was stimulated in the electrical stimulation-induced AAR, while chemical stimulation of iWAT with capsaicin may activate all afferents of the adipose pat.

Electrical stimulation of eWAT afferent nerves caused a significant increase in RSNA but not in MAP. We previously found that bilateral baroreceptor denervation and vagotomy enhanced the MAP response to chemical stimulation of iWAT with capsaicin [10]. The result indicate that the AAR-induced pressor response is attenuated by baroreflex. It is known that baroreflex sensitivity is attenuated in hypertension [29]. We found that AAR was enhanced in obesity hypertension. The attenuated baroreflex may partially contribute to the enhanced AAR in obesity hypertension. A limitation in the present study is that the roles of GABA in the PVN in regulating the AAR was determined in rats under anesthesia after extensive surgery for PVN cannulation, RSNA recording and AAR evaluation. It is unclear whether the conclusion will apply to other animals or human being.

In summary, GABA in the PVN inhibits the AAR via both GABA_A_ and GABA_B_ receptors. GABA_A_ receptors in the PVN play a tonic role in inhibiting the AAR. Activation of GABA_A_ receptors in the PVN has a more potent inhibitory effect on the AAR than that of GABA_B_ receptors.

## Supporting Information

S1 ARRIVE Guidelines Checklist NC3Rs ARRIVE Guidelines Checklist (fillable). (PDF)

S1 Fig Electrical stimulation-induced AAR. The AAR was induced by stimulation (5V, 30Hz) of eWAT afferent nerves 4 times at an interval of 20 min in the same rat. Values are mean±SE. *P<0.05 vs. Control. n = 6.
(TIF)

S2 Fig Effects of microinjection of isoguvacine or baclofen into the PVN or AHA on AAR. The PVN microinjection of GABA_A_ receptor agonist isoguvacine (10 nmol) or a GABA_B_ receptor agonist baclofen (10 nmol) attenuated the AAR, while microinjection of same doses of isoguvacine or baclofen into the anterior hypothalamic area (AHA), which is adjacent to the PVN, had no significant effect on the AAR. Values are mean±SE. *P<0.05 vs. Saline. n = 3 for each group.
(TIF)
