# Supplementary figures and images for: GABA in Paraventricular Nucleus Regulates Adipose Afferent Reflex in Rats
Source: PLoS One. 2015 Aug 28;10(8):e0136983. doi: 10.1371/journal.pone.0136983 (PMC4552845; doi:10.1371/journal.pone.0136983)

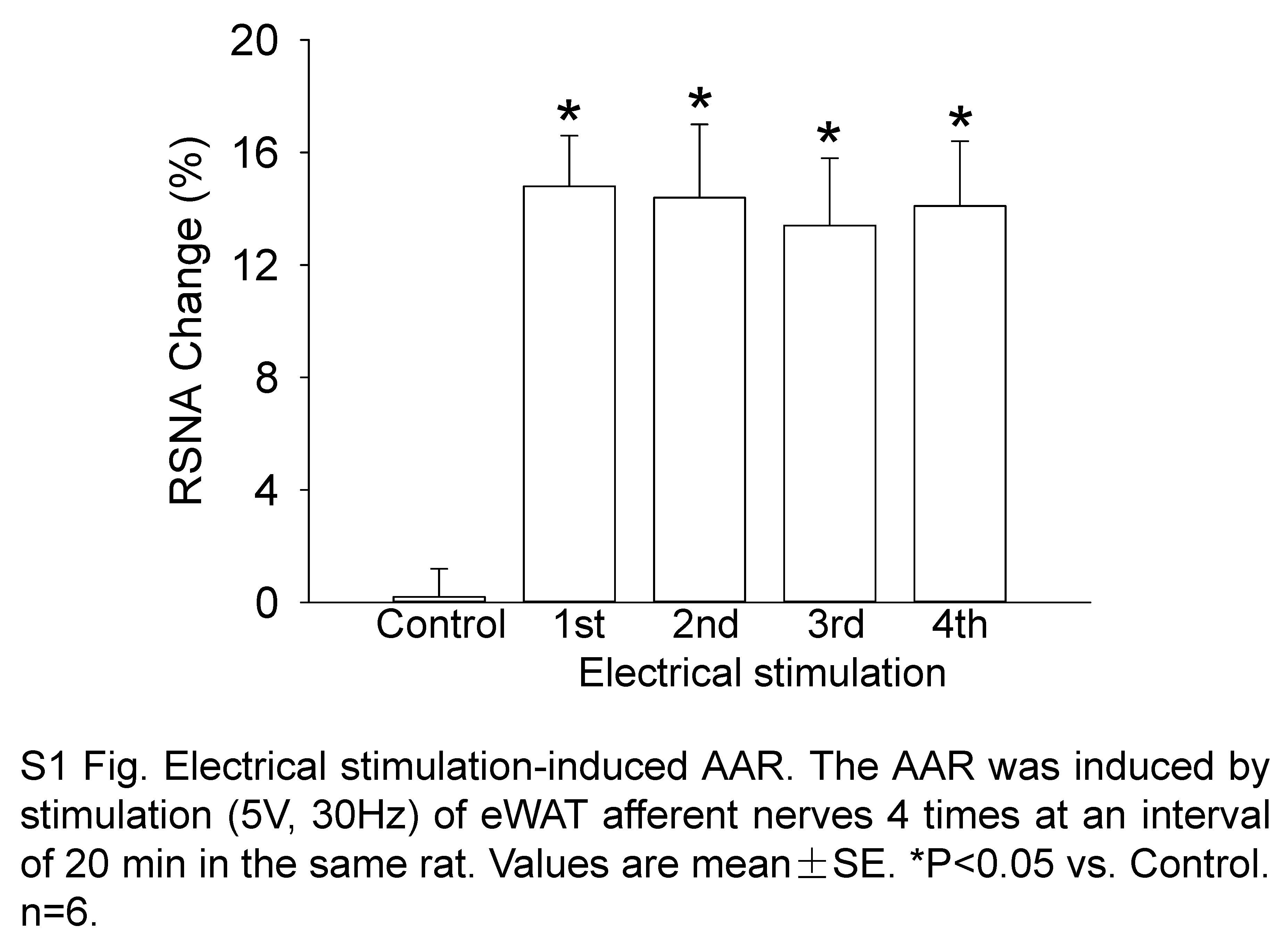

Supplement: S1 Fig — The AAR was induced by stimulation (5V, 30Hz) of eWAT afferent nerves 4 times at an interval of 20 min in the same rat. Values are mean±SE. *P<0.05 vs. Control. n = 6. (TIF) [file pone.0136983.s002.tif]

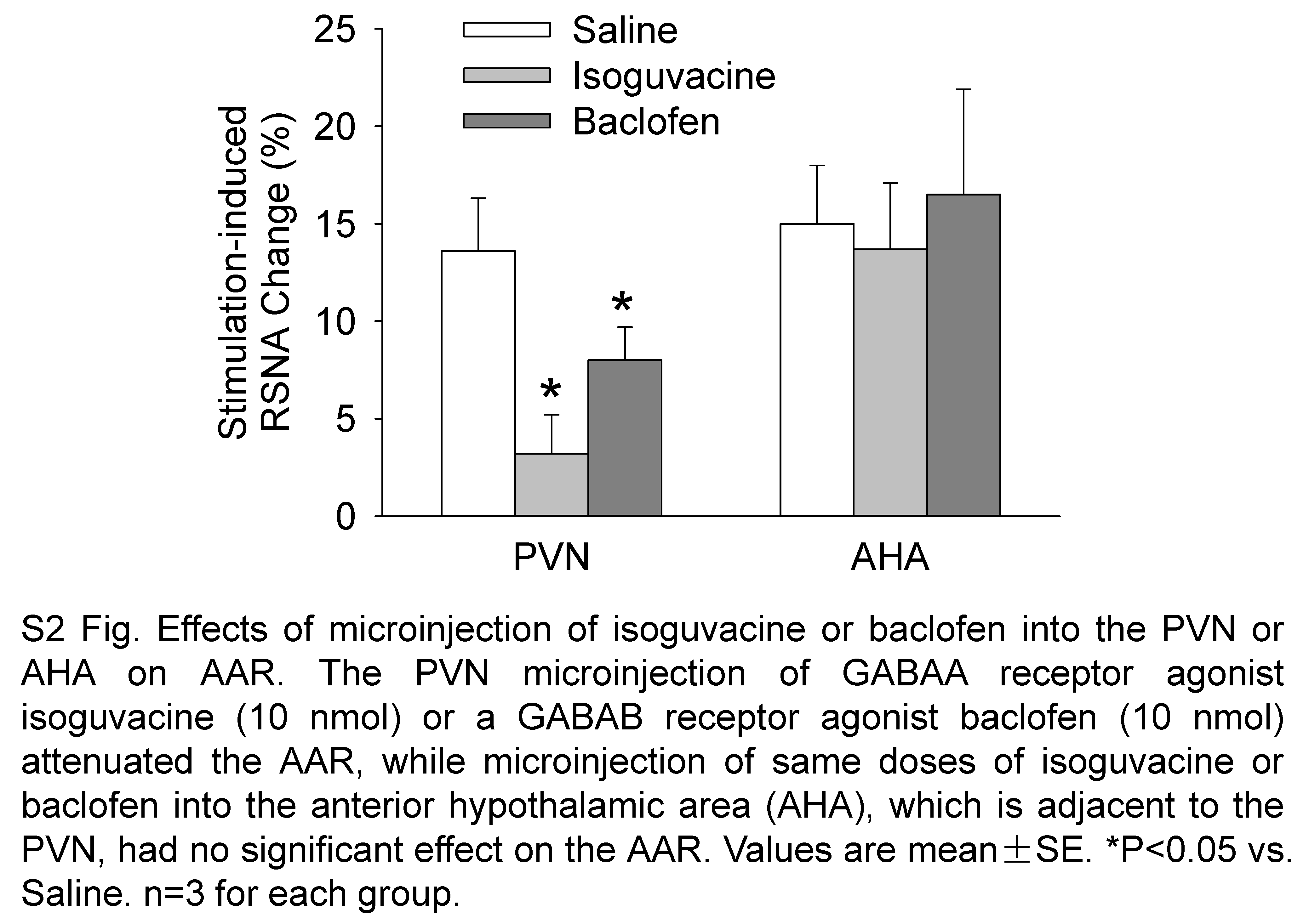

Supplement: S2 Fig — The PVN microinjection of GABAA receptor agonist isoguvacine (10 nmol) or a GABAB receptor agonist baclofen (10 nmol) attenuated the AAR, while microinjection of same doses of isoguvacine or baclofen into the anterior hypothalamic area (AHA), which is adjacent to the PVN, had no significant effect on the AAR. Values are mean±SE. *P<0.05 vs. Saline. n = 3 for each group. (TIF) [file pone.0136983.s003.tif]
